# Preparation of palladized carbon nanotubes encapsulated iron composites: highly efficient dechlorination for trichloroethylene and low corrosion of nanoiron

**DOI:** 10.1098/rsos.172242

**Published:** 2018-06-27

**Authors:** Xinyu Wang, Wei Wang, Greg Lowry, Xiaoyan Li, Yajie Guo, Tielong Li

**Affiliations:** 1 College of Environmental Science and Engineering/Tianjin Key Laboratory of Environmental Remediation and Pollution Control/Ministry of Education Key Laboratory of Pollution Processes and Environmental Criteria, Nankai University, Wei Jin Road 94, Tianjin 300071, People's Republic of China; 2 Department of Civil and Environmental Engineering, Carnegie Mellon University, Pittsburgh, PA 15213, USA

**Keywords:** carbon nanotube, trichloroethylene, hydrogen production by corrosion, nanoscale zerovalent iron, palladium

## Abstract

A method developed based on the capillary effect and capillary condensation theory was used to synthesize an innovative Fe/C/Pd composite in this study. This composite (Fe@CNTs@Pd) consists of carbon nanotubes (CNTs) with nanoscale zerovalent iron (NZVI) on the inner surface and palladium nanoparticles supported on the outer surface of CNTs. This structure successfully addresses the problems of high iron corrosion rate and lower utilization rate of hydrogen in the application of bimetal nanoparticles for trichloroethylene (TCE) removal. TCE degradation experiments and electrochemical tests were conducted to investigate the material properties and reaction mechanisms of the composite. It is found that the prepared composite material contribute a high level of TCE dechlorination rate and substantially reduced hydrogen production during iron corrosion in water compared with the conventional CNTs-supported bimetal materials (Fe/Pd@CNTs). Hydrogen spillover effect helps the reactivity of Fe@CNTs@Pd for TCE degradation and suppressed the galvanic cell effect, which results in a stronger resistance to corrosion. Although the *K*_obs_ of Fe@CNTs@Pd was 16.87% lower than that of Fe/Pd@CNTs, the hydrogen production rate of Fe@CNTs@Pd was 10 times slower than that of Fe/Pd@CNTs. Therefore, Fe@CNTs@Pd shows a significant reduction in the corrosion rate at a cost of slightly slower degradation of TCE. In sum, the prepared composites demonstrate important characteristics, including alleviating NZVI agglomeration, maintaining high TCE removal efficiency and reducing the corrosion of NZVI.

## 1. Introduction

Nanoscale zerovalent iron (NZVI) has been widely researched and applied in environmental remediation and wastewater treatment, especially for halohydrocarbon contamination, such as trichloroethylene (TCE) and chlorophenols [1–3]. The reaction rates, pathways, and factors influencing TCE degradation by NZVI were specific [4,5]. For example, Wang & Zhang synthesized nanoscale iron particles to dechlorinate TCE and polychlorinated biphenyls, and demonstrated that fast and complete removal is associated with the high surface activity of the NZVI [6]. However, NZVI presents a few limitations in the applications. An anaerobic corrosion of NZVI occurring in water is associated with the production of hydrogen [7]. It forms an oxide layer on the NZVI surface, and the generated hydrogen was adsorbed onto the surface which hinders further reaction with contaminants. This results in an enormous waste of NZVI and decreases the utilization rate of hydrogen. Additionally, some studies have shown the formation of toxic by-products (e.g. vinyl chloride) in TCE dechlorination process by zerovalent iron [8].

To improve the degradation rate of halogenated hydrocarbons and inhibit by-product formations, many researchers have engaged in preparing iron-based bimetal materials. Zhao and co-workers used carboxymethyl cellulose as a stabilizer to prepare Fe–Pd nanoparticles, which exhibited high reactivity for the degradation of TCE [9]. Within a bimetallic system, one metal (Fe) serves as the anode and the other metal (e.g. Pd) serves as the cathode due to the difference in metal potential. The formation of galvanic cell contributes to the increase in dechlorination reaction rate [10]. It is hypothesized that the galvanic cell formed between iron and a doped metal is vital for the generation of active hydrogen atoms and TCE degradation in the system [11]. Pairing palladium, as a reduction catalyst, with NZVI has been found to be the most effective combination for TCE degradation. The concentration of iron and dechlorination by-products (e.g. vinyl chloride, dichloroethylene) in palladized iron system is one order of magnitude lower than that in an iron system [12]. However, the increase in iron corrosion rate far exceeds the TCE reduction rate and the excess hydrogen gas cannot be fully used. In some conditions, TCE reduction rate increased by 11.5 times, while the corrosion rate of palladized iron jumped by 31.4 times [11].

The performance of supported nanoiron material has been well recognized due to its outstanding physical and chemical characteristics, such as the fast dispersion of nanoiron and the strong adsorption of contaminants [13]. Supported nanoiron is often prepared by using activated carbon as the carrier [14,15]. However, two metals in direct contact on the supporting material could still form a galvanic cell and cause excessive iron corrosion, especially when the iron is attached to a metal that has a higher reduction potential. Therefore, a method that can separate two metals is likely to help avoid the performance issues related to the bimetal materials.

Herein, a new method for synthesizing a bimetal material is presented. Using this new method, the effect of galvanic cell is weakened while the corrosion resistance is improved. Hydrogen spillover effect, which is defined as activated hydrogen atoms being generated on metal catalyst particles and then transferred to the adjacent surfaces of the supporting material via surface migration [16], has been well studied [17–19]. Hydrogen molecules can dissociate into activated hydrogen atoms rapidly on the surface of platinum (Pt), palladium (Pd) or other rare and precious metals, which can occur at room temperature and ambient pressures [20]. Therefore, a bifunctional material composed of a hydrogen-producing metal and a hydrogen-dissociating metal is expected to be an excellent catalyst to degrade TCE and other organic pollutants. The selection of appropriate supporting materials is critical to separate the two metals and support the migration of hydrogen. Discovered by Iijima in 1991, carbon nanotubes (CNTs) have many excellent properties, such as high conductivity, excellent hydrogen storage capability and small size effect [21–23]. CNTs are formed by one or up to dozens of graphitic shells that bend into a cylindrical tube seamlessly in the form of single-walled nanotubes and multi-walled nanotubes (MWNTs) [24]. It has been noted that between each layer of MWNTs there are apparent defects, which can be decorated by acid as active sites for metals. It is promising to use CNTs as a carrier due to their special structure. Researchers have attempted to fill CNTs with metals or metal oxides [25,26].

In this study, we propose a new approach to separate Fe and Pd on the CNTs. A MWNT is designed as a nanocapsule, which is filled with NZVI and covered by palladium nanoparticles. The wall of CNTs is used to separate the two metals (Fe and Pd). This synthesis method is grounded upon the capillary effect and capillary condensation theory. The general workflow of this method is that iron salt enters the tubes of CNTs first and hydrophobic solvent is then used to dissolve palladium salt and added to the CNTs which have been filled with water. This ensures that Pd is excluded from entering the inner tube of CNTs. Xylene is the ideal solvent for palladium salt due to its lower density than water and insolubility in water. Using the prepared nanocomposites, TCE degradation ratio has been found to be maintained at a high level, but the hydrogen production is dramatically decreased.

## 2. Material and methods

### 2.1 Materials and chemicals

Methanol (99.9%, HPLC grade, Concord), xylene (99%, Macklin), TCE (99%, Kermel), palladium acetate (Pd ≥ 47.4%, Mascot), iron nitrate nonahydrate (98.5%, Huaxueshiji Co., Tianjin), *trans*-dichloroethene (standard grade, AccuStandard) *cis*-dichloroethene (standard grade, AccuStandard), 1,1-dichloroethene (standard grade, AccuStandard), vinyl chloride (2 g l^−1^ in methanol, AccuStandard) and CNTs (Chengdu Organic Chemicals Co., Ltd, Chinese Academy of Sciences) were purchased from commercial sources in China and used as received. The chemicals are analytical grade reagents and used without any further purification unless otherwise specified. All the reagents were prepared with deionized water.

### 2.2 Preparation of composites

**Figure 1.**
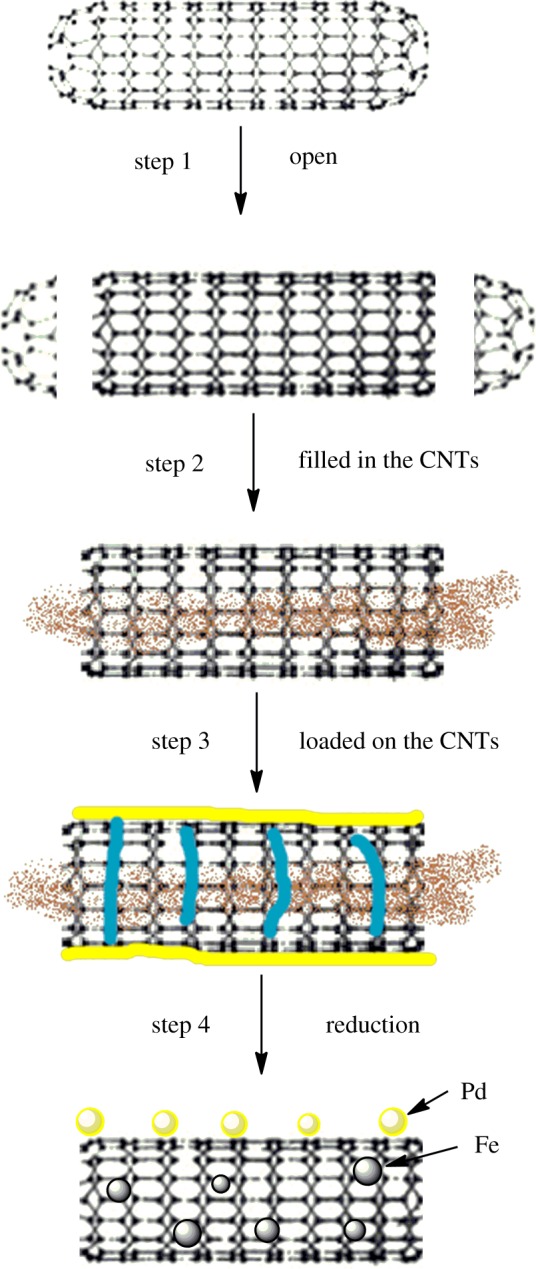
Preparation flow diagram of Fe@CNTs@Pd.

Multi-walled CNTs with an outer diameter ranging from 8 to 15 nm were used as supporting materials to separate the two metals (iron and palladium). There are four steps in the preparation of Fe@CNTs@Pd composites, as described in figure 1. Step 1 is to purify and open up the CNTs. A certain amount of CNTs were suspended in concentrated nitric acid (mass ratio of CNTs and HNO_3_ was 1 : 70) and refluxed for 14 h in an oil bath at 120–140°C. After washing acid treated CNTs with deionized water (until neutral pH = 6–7) and drying at 70°C overnight, the purified and opened CNTs were obtained. The weight loss in step 1 was less than 5%. The second step is to load ferric salt into the CNTs (Fe salt@CNTs). Certain concentration of iron nitrate nonahydrate solution was added to the opened CNTs and treated with ultrasound for 8 h, then the mixture was stirred until dry at room temperature. After treatment in boiling nitric acid, there are many defects inside of CNTs, and a large number of iron ions can enter the CNTs through capillary condensation. Theoretically, iron content (mFe/m(CNTs + Fe)) in the composite should be 30%. The mixture was scraped off, ground and then washed with deionized water and dried at 70°C overnight. The iron ions absorbed onto CNT outer surfaces can be removed after washing. The third step is to load palladium salt (Fe/Pd salt@CNTs) onto the outer surface of CNTs. Xylene was used as a solvent in this step and 500 mg l^−1^ solution of palladium salt was prepared. The Fe salt@CNTs was added to a beaker, and water was added by drops just to immerse the CNTs, then palladium acetate–xylene solution was added. The mixture was dried at 70°C overnight. The hydrophobicity and the lower specific gravity of xylene ensured that palladium ions could not enter into the CNTs. Theoretically, the Pd content (mPd/m(Fe salt@CNTs)) should be 0.1%. Step 4 is to pyrolyze and reduce metal salts. The Fe/Pd salt@CNTs was heated to 600°C at a rate of 5°C min under Ar–H_2_ gas mixture (H_2_ volume fraction of 5%) and maintained at 600°C for 3 h. The flow rate of the gas was 100 ml min^−1^. The prepared sample was cooled to ambient temperature under Ar–H_2_ gas. The generated particles were denoted as Fe@CNTs@Pd.

For comparison, Fe/Pd@CNTs, in which Fe and Pd were not separated on CNTs, were obtained via a similar process mentioned above without the step of adding water to immerse CNTs. Palladium acetate–xylene solution was added to the beaker containing Fe salt@CNTS directly. Blank CNTs were prepared just following steps 1 and 4.

### 2.3 Sample characterization

The power X-ray diffraction (XRD) analysis was performed to study the phase of samples using a Rigaku Ulitma IV X-ray diffractometer equipped with a Cu K*α* X-ray source. These XRD tests were conducted from 10° to 90° with a step size of 10°min^−1^. X-ray photoelectron spectroscopy (XPS) analysis was carried out to study the valence state of iron loaded inside the CNTs. The source of X-rays was Al K*α* with a quartz monochrome. The binding energy scale was calibrated using the graphitic carbon C1s feature at 284.0 eV. The N_2_ adsorption and desorption isotherms were determined with a Micromeritica ASAP 2460 instrument. BET (Brunauer–Emmett–Teller) surface areas were estimated based on a relative pressure range from 0.05 to 0.3 in the BET mode. Pore size distribution was analysed using the Barrett–Joyner–Halenda methods from the adsorption branch of the isotherm. High-resolution transmission electron microscopy (HR-TEM) was conducted using a Tecnai G2 F20 microscope. Small quantities of powdered samples were dispersed in deionized water, and a 200-mesh Cu grid with a Lacey-carbon support film dipped into the suspension was dried.

The Fe and Pd contents of the samples were extracted through mixed acid digestion (V(H_2_SO_4_ : HNO_3_) = 3 : 1) and determined by an Analytikjena Contra 700 continuum light source atomic absorption spectrophotometer. The Fe^0^ content of the samples was measured through hydrochloric acid digestion in a closed container with headspace. As H_2_ is produced with reaction between Fe^0^ and hydrochloric acid, the H_2_ amount was used to quantify the Fe^0^ content of the samples.

### 2.4 Degradation of trichloroethylene and product analysis

Batch experiments were conducted to test the reactivity of the composites for the dechlorination of TCE. Of note, 0.5 g freshly prepared Fe@CNTs@Pd powders and 100 ml of 20 mg l^−1^ TCE were added into a 250 ml reaction bottle with a Teflon Mininert valve. The bottles were then mounted in a rotary shaker (180 r.p.m.) at room temperature (25°C). The content of residual TCE, produced hydrogen and degradation products were monitored by headspace gas chromatography (GC) analysis. For the headspace samples, 0.2 ml of headspace gas was introduced by splitless injection into the GC at a 200°C injector temperature. All chlorinated ethenes (including TCE, *trans*-dichloroethene, *cis*-dichloroethene, 1,1-dichloroethene, chloroethene) were analysed by an Agilent 6820 GC equipped with a flame ionization detector (FID) and a 30 m HP-5MS capillary column. Ethane, ethylene and ethyne were analysed by an Agilent 6820 GC equipped with an FID and a 30 m HP-PLOT Q capillary column. H_2_ was analysed by the GC with thermal conductivity detector and a 2 m Agilent-5A packed column. Similar experiments were also applied to the blank CNTs and Fe/Pd@CNTs as controls. The evolution of chloride ion concentration was also measured using a DIONEX IC-5000 ion chromatograph.

### 2.5 Electrochemical test

To study electronic transmission performance of the composites, a series of electrochemical tests were carried out. Electrochemical impedance spectroscopy (EIS) was used to study the resistance of the composites in reaction systems. Linear sweep voltammetry (LSV) was used to analyse the polarization degree of the composites. Tafel measurement was used to track the electron transfer speed.

The addition of 1 g Fe@CNTs@Pd to a suitable amount of anhydrous ethanol and ultrasonic agitation was conducted for 30 min. After that, 0.5 g polytetrafluoroethylene was mixed into the solution and subjected to ultrasonic stirring for 30 min. Then, the mixture solution was heated and stirred in 80°C water bath until it formed a paste. Finally, the formed paste was rolled onto a nickel foam as a working electrode. Similarly, blank CNTs and Fe/Pd@CNTs electrodes were prepared based on the above method.

EIS, LSV and Tafel measurements of all the anodes were conducted with a CHI 660E electrochemical workstation (Shanghai Chen Hua Instrument) using a three-electrode system. Prepared electrodes served as working electrodes, saturated calomel electrode was used as the reference electrode, and a platinum plate electrode works as the auxiliary electrode. EIS was performed at a frequency range from 100 kHz to 10 MHz with an amplitude of 10 mV. Tafel curve was measured by sweeping from overpotential of 0 V to 200 mV at a scan rate of 1 mV s^−1^.

## 3. Results and discussion

### 3.1 Characterization of samples

**Figure 2.**
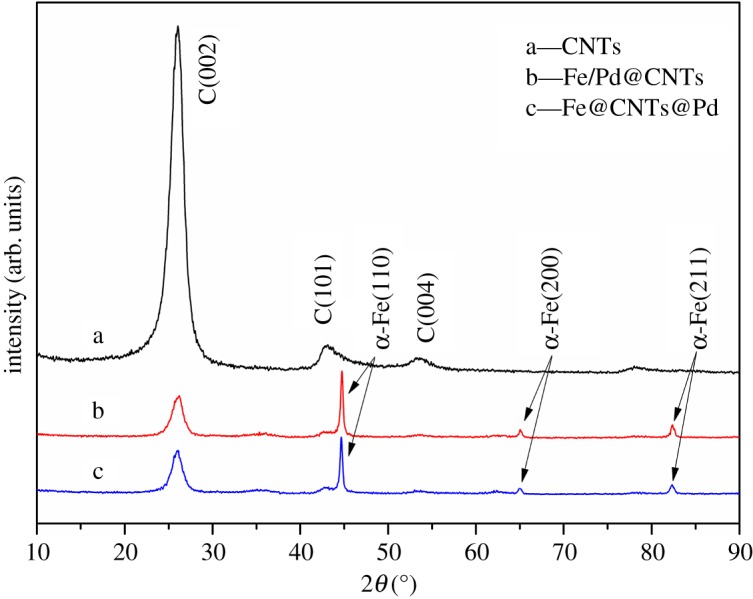
XRD patterns of blank CNTs, Fe/Pd@CNTs and Fe@CNTs@Pd.

Figure 2 shows XRD patterns of the blank CNTs, Fe/Pd@CNTs and Fe@CNTs@Pd. The strongest peak of curve *a* at 2*θ *= 26.3° is assigned to (002) crystal face of graphite carbon (JCPDS card No. 41-1487). Few differences were observed between curves *b* and *c*, which represent Fe/Pd@CNTs and Fe@CNTs@Pd, respectively. This may be explained by that the Pd content is scarce and below the detection limit of this instrument. The strongest peak of composites at 2*θ *= 44.6° is assigned to (110) crystal face of α-Fe (JCPDS card No. 06-0696), and weak peaks corresponding to other crystal faces of α-Fe were also observed. It is noted that the peak intensity of graphite carbon significantly decreased after the Fe loading. This indicates that the Fe nanoparticles have been loaded on CNTs [27]. Similar results were also reported in other studies [28].

**Figure 3.**
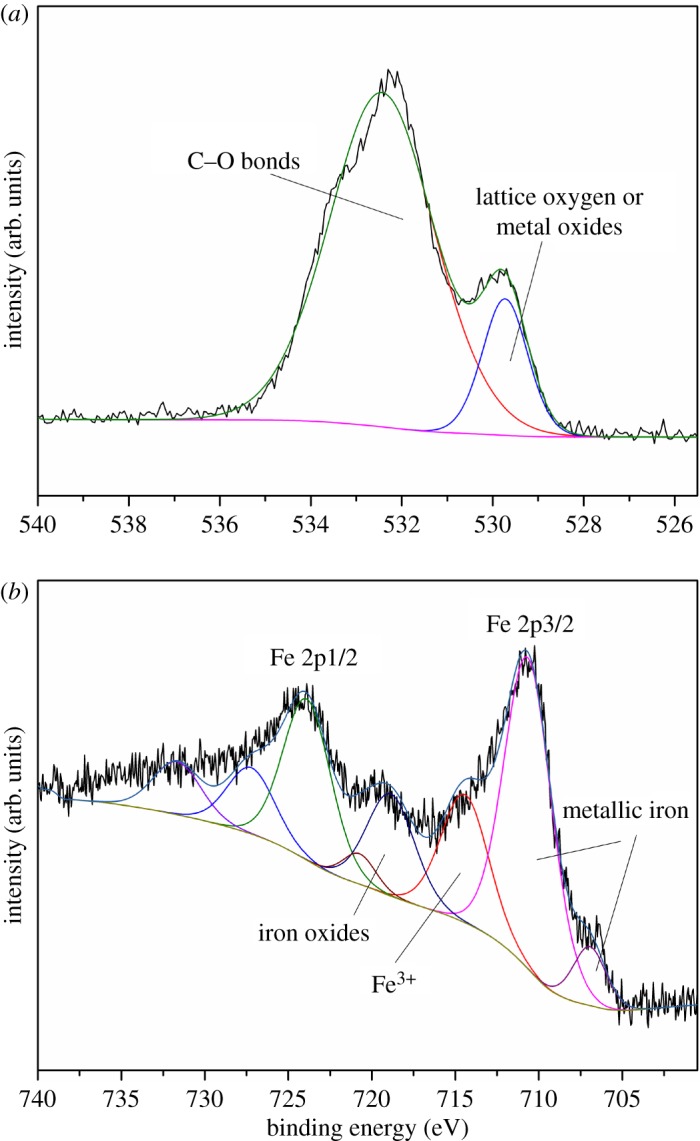
XPS spectra: O1s high-resolution spectrum of Fe@CNTs@Pd (*a*) and Fe 2p high-resolution spectrum of Fe@CNTs@Pd (*b*).

To further investigate the elemental chemical state in Fe@CNTs@Pd, XPS measurement was performed. The spectrum of full range scan indicated that C, O and Fe elements were the major elements in the composite (electronic supplementary material, figure S1a). Pd was not detected in the spectrum owing to its low level. C1s XPS spectrum is presented in electronic supplementary material, figure S1b, and three peaks were fitted by means of peak-differentiating analysis. The major peak at 284.1 eV was characteristic peak of graphite carbon, which was consistent with the results of other studies [29]. The peak at 284.8 eV was assigned to C–C bond, which was caused by adventitious carbon contamination and/or sp^3^ carbon existing in composites. The peak of 289.1 eV was mainly caused by the oxygenic groups introduced into the composites. Figure 3*a* displays the XPS spectrum of O1s, which was deconvoluted into two peaks corresponding to different chemical states of oxygen atoms. The main peak was located at 532.4 eV, which corresponds with the C–O bond [30,31]. Another peak at 529.7 eV was generally considered to be caused by lattice oxygen or metal oxides. Small amounts of Fe were oxidized during the XPS test [32,33]. Figure 3*b* shows Fe 2p XPS spectrum, which can be deconvoluted into four couples of peaks. The peaks of 2p3/2 at binding energy near 710.6 and 706.9 eV were observed, indicating zerovalent iron was the main chemical state of Fe element [34,35]. Other peaks correspond to iron oxidation state caused by the oxygen environment mentioned above. The results of XPS revealed that oxygenic groups exist in the composites, and Fe^0^ particles were loaded on the CNTs successfully.

Table 1 shows the BET specific surface areas of different materials. It is clear that nitric acid-activated CNTs can enlarge the specific surface area by 38.7% compared with the unmodified CNTs. The specific surface area decreased after metal nanoparticles were loaded on CNTs, because metals filled up the pores of CNTs [36]. All the materials exhibited a type IV isotherm, which has significant hysteresis loop (electronic supplementary material, figure S2). The corresponding pore size distributions are shown in electronic supplementary material, figure S2a–d. Macropores and mesoporous are rich in unmodified CNTs. Some pores can be produced on the defect areas of CNTs through nitric acid oxidation and mesopores form in this process (electronic supplementary material, figure S2a,b). The common pore size of CNTs becomes smaller after nitric acid modification and coating metals. Most common pore diameter of blank CNTs is about 40 nm, which is caused by defects between the layers of MWNTs. The pore diameters of Fe/Pd@CNTs and Fe@CNTs@Pd are about 20 nm, indicating iron occupying the reactive sites. According to the results of BET test, the composites could have relatively strong adsorption capacity.

**Table 1.** BET specific surface areas and most probable pore diameter of different materials.

materials	BET specific surface area (m^2 ^g^−1^)	most probable pore diameter (nm)
unmodified CNTs	173.7805	>70
blank CNTs	241.0275	40
Fe/Pd@CNTs	216.9462	20
Fe@CNTs@Pd	226.9593	20

**Figure 4.**
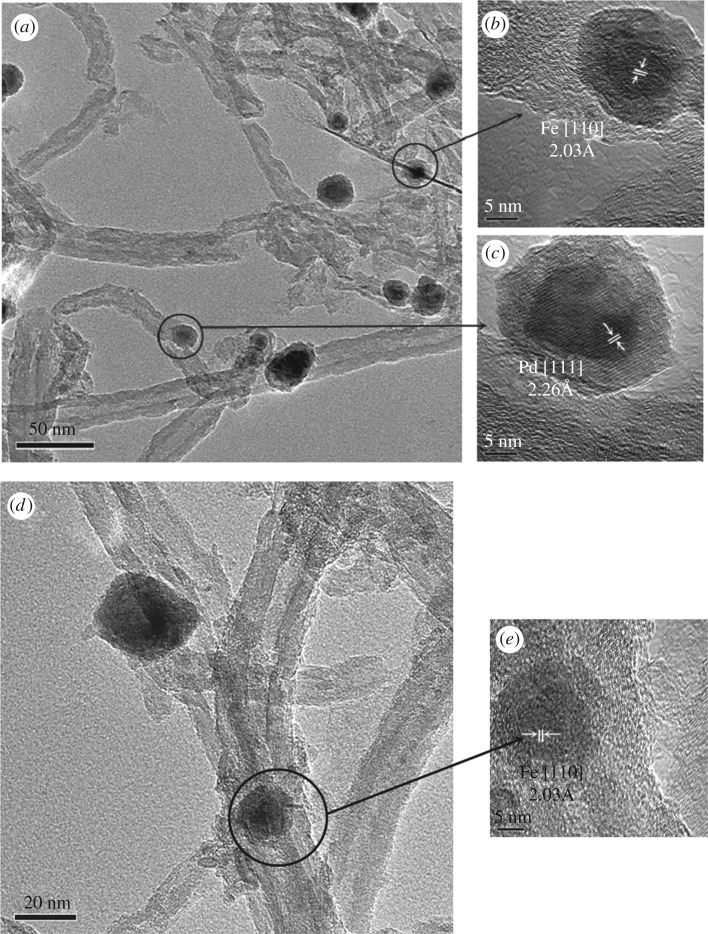
HR-TEM images of Fe@CNTs@Pd (*a, d*), inner particles of CNTs (*b, e*), surface particles of CNTs (*c*).

Figure 4 displays HR-TEM images of Fe@CNTs@Pd. Particles with uniform size of 10–20 nm can be observed, and the 2.03 ± 0.05 Å d spacing corresponding to the [110] planes of iron was observed inside the CNTs (figure 4*b*,*e*). Surface particles of CNTs have 2.26 ± 0.05 Å d spacing corresponding to the [111] planes of Pd (figure 4*c*). These results indicated that Fe and Pd were well separated using the above method.

The Fe and Pd contents in the two composites were determined through digesting the composites, and the experiment was repeated three times. The contents of Fe and Pd are stable under the preparation conditions in three experiments with average contents of Fe and Pd in Fe@CNTs@Pd at 10.76% and 0.07%, respectively.

Fe^0^ contents in Fe/Pd@CNTs and Fe@CNTs@Pd were 9.86% and 10.05%, respectively. The results were slightly lower than the Fe content, which may be caused by two reasons. First, some hydrogen is adsorbed on the CNTs or dissolves in the water, which causes a reduction of hydrogen amount in the headspace of closed container. Second, a fraction of Fe^0^ particles is oxidized. The entire analysis results have indicated that the Fe^0^ content is about 10% in this preparation method and only very small amounts of Fe^0^ may be oxidized.

### 3.2 Batch experiments for trichloroethylene degradation

#### 3.2.1 Trichloroethylene degradation and hydrogen production analysis

**Figure 5.**
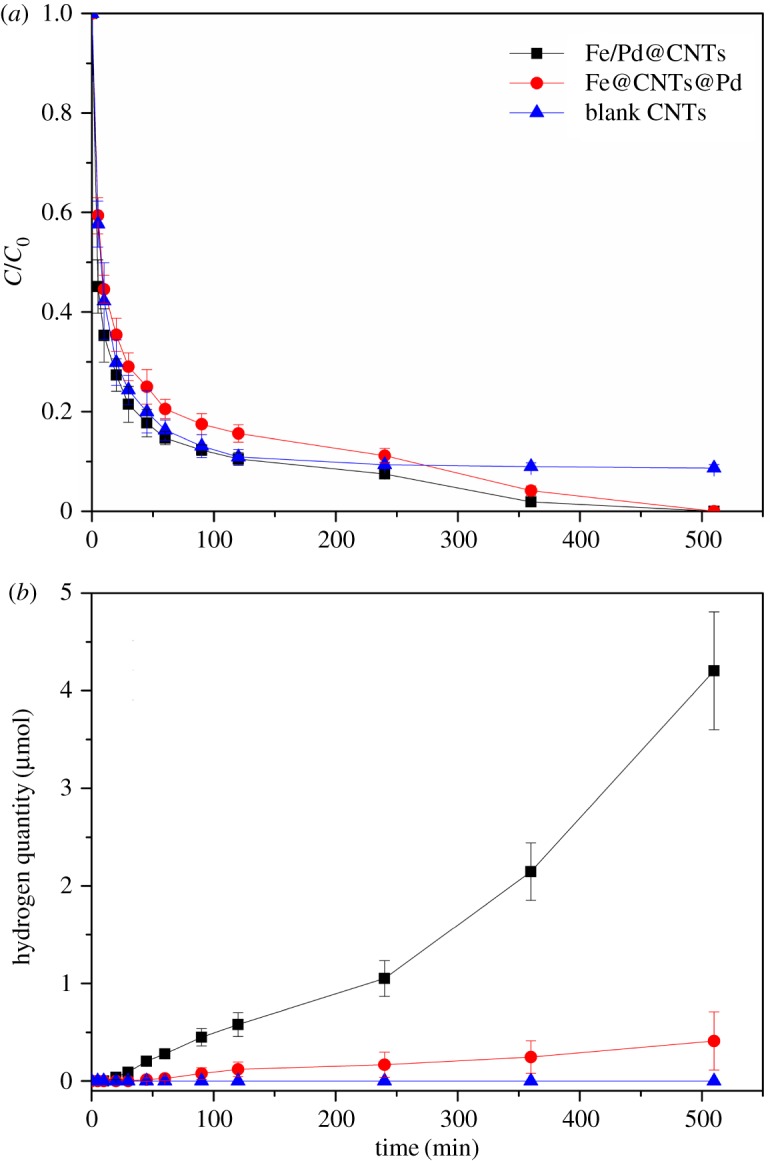
TCE removal by blank CNTs, Fe/Pd@CNTs and Fe@CNTs@Pd (*a*). Hydrogen quantity in the 20 mg l^−1^ TCE system (*b*).

Figure 5*a* presents TCE removal by blank CNTs, Fe/Pd@CNTs and Fe@CNTs@Pd. Blank CNTs remove TCE in the water only through the adsorption effect. After reaching absorption equilibrium in two hours, TCE concentration stops its decline and stays stable. However, the removal rates of TCE by Fe@CNTs@Pd and Fe/Pd@CNTs are fast and the TCE cannot be detected after 500 min. The plots of Fe@CNTs@Pd and Fe/Pd@CNTs in figure 5*a* were fitted with the pseudo-first-order rate equation:

$$-\frac{\text{d}c}{\text{d}t}=K_{\mathrm{obs}}C,$$

where *K*_obs_ is the observed pseudo-first-order rate constant (min^−1^), *C* is the concentration of aqueous phase TCE (mg l^−1^). The fitting results are presented in table 2.

**Table 2:** The pseudo-first-order model fitting parameters of Fe/Pd@CNTs and Fe@CNTs@Pd.

	*K*_obs_ (min^−1^)	*t*_1/2_ (min)	*R*^2^
Fe/Pd@CNTs	0.0083	83.51	0.81298
Fe@CNTs@Pd	0.0069	100.46	0.81178

The value of *K*_obs_ decreased by only 16.87% when the metals of Fe and Pd were separated on the CNTs. The degradation rate of TCE was exceedingly fast for Fe/Pd@CNTs. The main reason is that the galvanic effect can accelerate the electron transfer. Some of these electrons react with water quickly to produce a large quantity of hydrogen, which has a strong impact on the degradation processes of TCE. Many researchers believe that hydrogen, especially active hydrogen atoms, can serve as effective electron donors [11]. Therefore, Fe/Pd@CNTs and Fe@CNTs@Pd can degrade TCE at a speedy rate regardless of whether Fe and Pd were separated by CNTs or not. In addition to the reaction with TCE, the adsorption of Fe@CNTs@Pd dominates the initial stage of reaction. The adsorption capacity of CNTs is strong and the adsorptive rate of TCE reached 90% in 2 h. The pseudo-second-order model was used to fit the process of removing TCE, which can be represented by the following equation:

$$\frac{t}{Q_{t}}=\frac{1}{k_{2}Q_{e}^{2}}+\frac{t}{Q_{e}},$$

where *Q*_e_ is the amount of removed TCE (including adsorption and degradation) at equilibrium time (mg g^−1^), *Q_t_* is the amount of TCE removed at sampling time (mg g^−1^), and *k*_2_ is the pseudo-second-order adsorption rate constant (min^−1^).

The reaction process represented by the data was fitted well by a pseudo-second-order reaction equation instead of a pseudo-first-order equation, implying the kinetics include an initial fast phase and a final slow phase. The *k*_2_ values of Fe/Pd@CNTs and Fe@CNTs@Pd were substantially lower than that of the blank CNTs, because iron nanoparticles occupy pore spaces of CNTs and absorption capacity of composites declines. The *Q*_e_ and *k*_2_ values are close for both Fe/Pd@CNTs and Fe@CNTs@Pd, indicating the TCE degradation is less affected regardless of whether Fe and Pd were separated by CNTs or not.

Figure 5*b* shows the generated hydrogen quantity during the TCE degradation process. The amount of hydrogen in the Fe/Pd@CNTs system could reach up to 100 µl, which was 10 times higher than the peak in the Fe@CNTs@Pd system. The average production rate of hydrogen was about 0.18 µl min^−1^ for Fe/Pd@CNTs, which was 10 times higher than that for Fe@CNTs@Pd. It is obvious that Fe/Pd@CNTs can slightly improve the degradation of TCE, but significantly boost the corrosion of Fe. Therefore, it is valuable to separate the two metals, because the corrosion of NZVI in Fe@CNTs@Pd is remarkably suppressed but with the TCE degradation staying at a high level.

#### 3.2.2 Products analysis of trichloroethylene degradation

**Figure 6.**
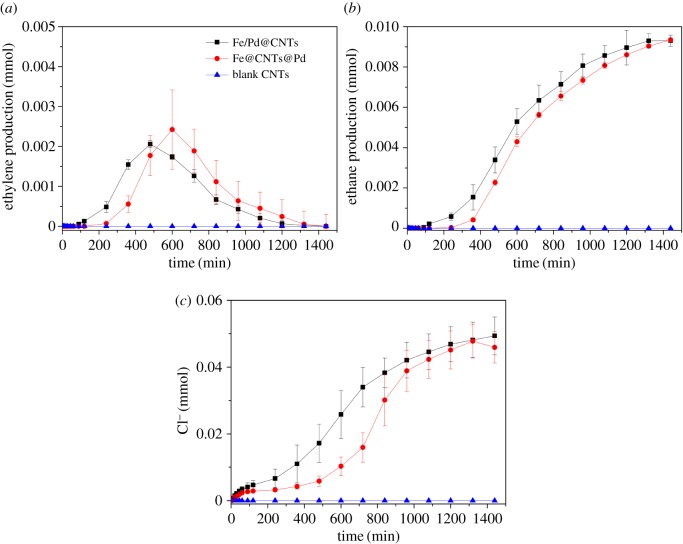
(*a*) Ethylene production, (*b*) ethane production and (*c*) evolution of chloride ion concentration in 20 mg l^−1^ TCE systems treated by blank CNTs, Fe/Pd@CNTs and Fe@CNTs@Pd.

The degradation pathways of TCE treated by Fe–Pd system have been widely studied. Similar to the Fe–Pd bimetal systems [12,37], no chlorinated intermediates (dichloroethylene, vinyl chloride) were detected in our conditions. Ethane and ethylene are degradation products and the results are shown in figure 6.

The evolution trends of ethylene and ethane production are consistent, which indicate the degradation pathways of TCE treated by Fe/Pd@CNTs and Fe@CNTs@Pd may be similar (figure 6*a*,*b*). Differences in conversion rates and yields exist between both products (ethylene, ethane). Ethylene and ethane are intermediate and end product, respectively, for Fe–Pd systems used for degrading TCE. These products are not detected in blank CNTs. The rate of ethane production for Fe@CNTs@Pd is slightly below that for Fe/Pd@CNTs, and the results agree with the above experiment of TCE degradation. Similarly, evolution of chloride ion concentration in the reaction systems indicates that Fe–Pd systems can dechlorinate TCE, which exist in the form of chloride ion in the systems (figure 6*c*). The dechlorination rate of TCE for Fe@CNTs@Pd decreased lightly compared with Fe/Pd@CNTs. Ultimately, the Fe/Pd@CNTs and Fe@CNTs@Pd in the ethylene, ethane and chloride ion are about equal.

**Figure 7.**
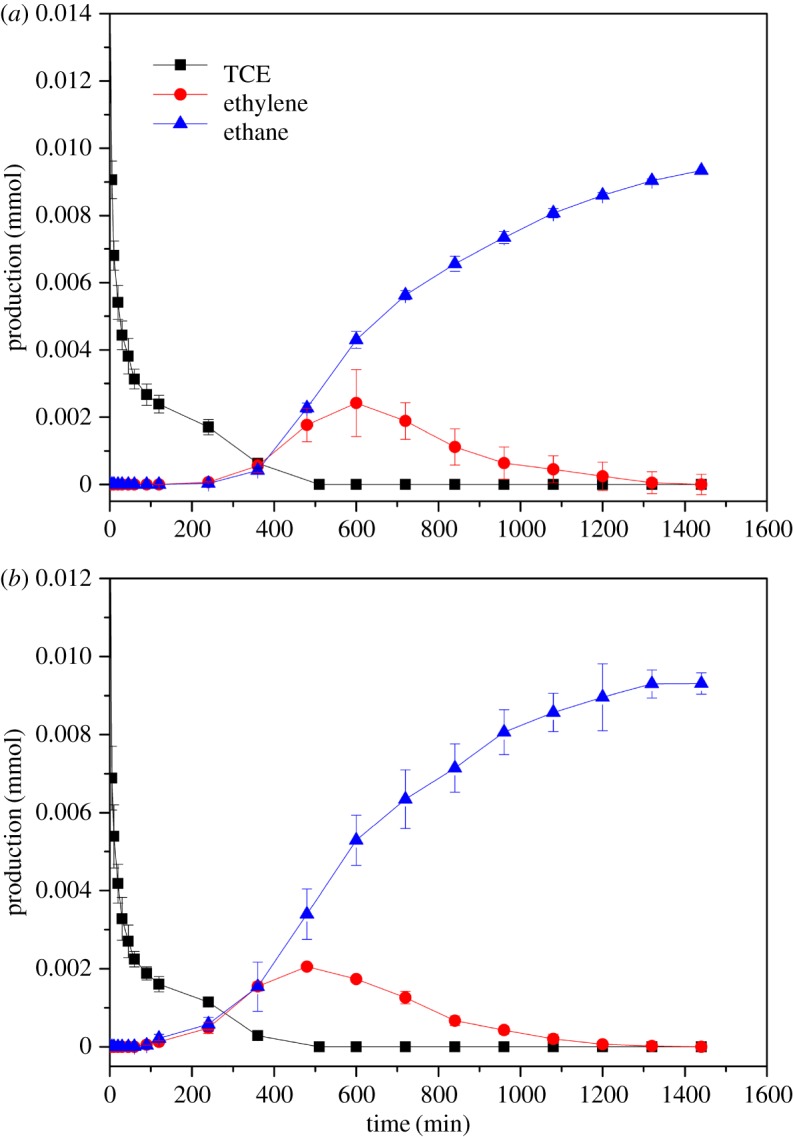
Ethylene production, ethane production and residual amount of TCE for Fe@CNTs@Pd (*a*) and Fe/Pd@CNTs (*b*) in 20 mg l^−1^ TCE systems.

Figure 7*a,b* shows that Fe@CNTs@Pd could degrade TCE rapidly and transform TCE into non-toxic and harmless ethane. According to the mass balance check, Cl is about 0.045 mmol in the end. This indicated TCE was degraded completely by Fe@CNTs@Pd or Fe/Pd@CNTs. Final detectable 67% of initial TCE content was transformed into ethane. 90% of TCE was quickly adsorbed by CNTs in the initial stage of reaction, along with 20% ethylene generated in the stage. In the end, no TCE and ethylene were detected and 67% ethane was detected in end-products. The remaining 33% end-products were not detected from the absorption of the composite material. The average generation rate of hydrogen due to corrosion is decreased 10 times when Fe and Pd were separated by CNTs, indicating the excellent performance of Fe@CNTs@Pd.

### 3.3 Mechanism study by electrochemical methods

#### 3.3.1 Linear sweep voltammetry polarization curves of the anodes

**Figure 8.**
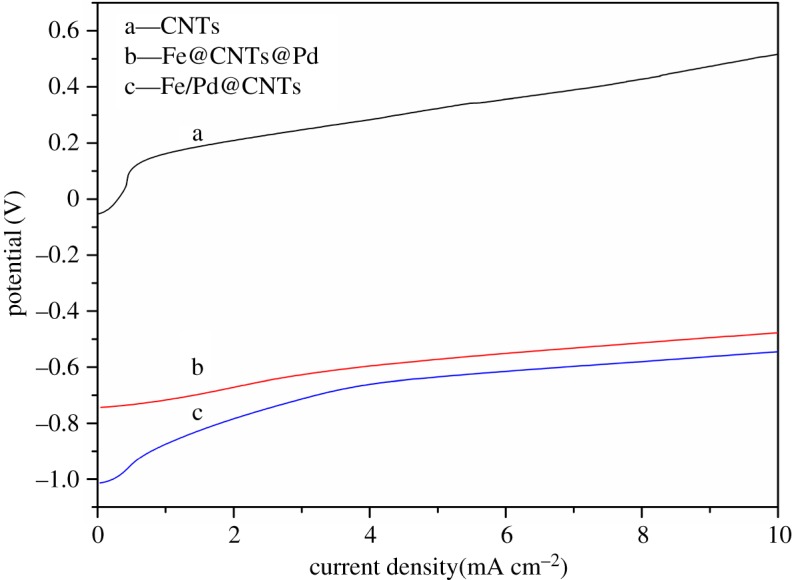
LSV anode polarization curves of the blank CNTs, Fe/Pd@CNTs, and Fe@CNTs@Pd.

The LSV polarization curves of the three materials are shown in figure 8. The slope of each curve is considered to represent the degree of polarization. The order of slope is: Fe/Pd@CNT≈Fe@CNTs@Pd<blank CNTs. The polarization is more serious and the reaction resistance of material is larger when the curve becomes steeper [38]. The polarization degree of blank CNTs was much higher than that of Fe@CNTs@Pd and Fe/Pd@CNTs, illustrating that the resistance of blank CNTs was much higher than that of Fe@CNTs@Pd and Fe/Pd@CNTs in the reaction. The impacts of Fe@CNTs@Pd and Fe/Pd@CNTs on the TCE degradation turned out to be small, which was consistent with the TCE degradation results.

#### 3.3.2 Electrochemical impedance spectroscopy of the anodes

**Figure 9.**
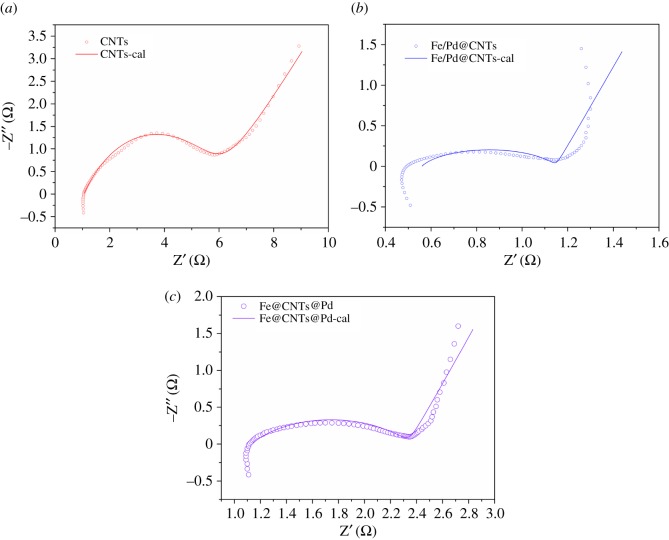
Nyquist plots of EIS fitting analysis of blank CNTs (*a*), Fe/Pd@CNTs (*b*), Fe@CNTs@Pd (*c*).

EIS has been used to assess the reaction performance and electron transfer kinetics of different anodes [39]. The EIS results of all the materials are displayed in figure 9. The ohmic resistance (*R*_O_), contact resistances (*R*_d_), charge transfer resistance (*R*_ct_) were calculated with the equivalent circuit as shown in table 4.

**Table 3.** The pseudo second-order model fitting parameters of Fe/Pd@CNTs and Fe@CNTs@Pd.

	*Q*_e_ (mg g^−1^)	*k*_2_(min^−1^)	*R*^2^
Fe/Pd@CNTs	4.02	0.0291	0.9987
Fe@CNTs@Pd	4.00	0.0193	0.9969
CNTs	3.69	0.0515	0.9998

Table 4 presents the fitting results of different materials_,_ indicating that total resistances after metals loading on CNTs showed an obvious decrease. Contact resistances of three materials are generally close with the exception of charge transfer resistance. Charge transfer resistances are decreased by 87.86% for Fe/Pd@CNTs and 75.02% for Fe@CNTs@Pd compared with CNTs, which illustrated electron transfer rate increased by loading metals on CNTs. Charge transfer resistance of Fe/Pd@CNTs is the lowest, and this caused the rapid reaction in liquid system. Fe/Pd@CNTs can produce larger amount of hydrogen through quick reaction with the water owing to fast electron transfer. Actually, the high electron transfer rate prohibits the hydrogen from being fully used. Fe@CNTs@Pd can help the controlled release of hydrogen with decreased charge transfer resistance. Compared with CNTs and Fe@CNTs@Pd, the ohmic resistance dropped by about 50% because Fe in the Fe/Pd@CNTs was quickly corroded and released into the water. These results also illustrated that Fe@CNTs@Pd has excellent corrosion resistance in liquid. The EIS results were consistent with the above hydrogen generation results.

**Table 4.** Electrochemical impedance fitting results of different materials based on the equivalent circuit.

	blank CNTS	Fe/Pd@CNTs	Fe@CNTs@Pd
*R*_O_ (Ω)	1.053	0.560	1.132
*R*_ct_ (Ω)	4.859	0.590	1.214
*R*_d_ (Ω)	0.495	0.556	0.612
total resistance (Ω)	6.407	1.706	2.958

#### 3.3.3 Tafel curves of the anodes

**Figure 10.**
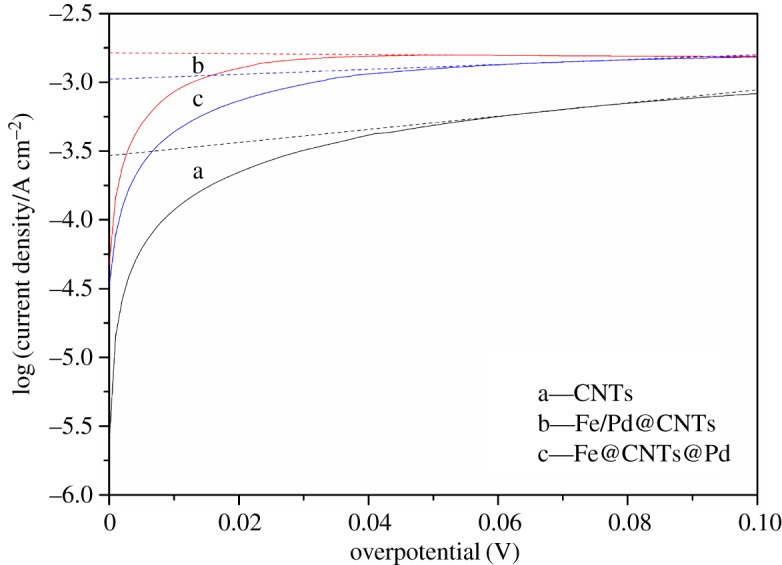
Tafel plots of blank CNTs, Fe/Pd@CNTs and Fe@CNTs@Pd.

Figure 10 shows the Tafel curves of different materials. The three curves exhibit a linear trend after a steep increase at the beginning. A linear Tafel regression (*R*^2 ^> 0.99) exists in the overpotential interval of 60–80 mV and the fitting results are presented in table 5. Exchange current density describes the reaction kinetics and activation energy [40]. Fe/Pd@CNTs had the highest *i*_0_ at 1.4488 × 10^−3 ^A cm^−2^, which was 4.2 times and 0.4 times higher than the blank CNTs (0.1595 A cm^−2^) and Fe@CNTs (0.5830 A cm^−2^), respectively. This implies that Fe/Pd@CNTs had faster reaction with lower activation barrier to TCE and water. The increase of activation barriers can reduce the rate of hydrogen production and avoid the waste of hydrogen. This shows that the design of iron loading on the inner wall of the CNTs and the loading of palladium on the outer wall of the CNTs can solve the problem of excessive corrosion of materials and significantly reduce the rate of hydrogen production. It can also maintain high TCE degradation rate. These results agree with the above analysis of LSV and EIS.

**Table 5.** Exchange current density calculated from the Tafel plots.

	fitted equation	*R*^2^	10^−3^*i*_0_ (A cm^−2^)
blank CNTs	*y* = −3.5337 + 4.7873*x*	0.9977	0.2794
Fe/Pd@CNTs	*y *= −2.8390 + 0.7507*x*	0.9906	1.4488
Fe@CNTs@Pd	*y *= −2.9774 + 1.7825*x*	0.9931	1.0534

## 4. Conclusion

Fe@CNTs@Pd composites were successfully prepared using a new technical design, in which NZVI was filled into the pores of CNTs and palladium nanoparticles were loaded on the outer surface of CNTs. This composite has exhibited excellent performance in TCE degradation and Fe corrosion prevention in aqueous system compared with Fe/Pd@CNTs. The separation of Fe salt and Pd salt on the CNTs played a significant role in achieving the goal of this design. Capillary condensation theory and concentration diffusion ensured that most Fe^3+^ ions were adsorbed into the inner tube of the CNTs in the process of iron loading. The hydrophobicity and lower specific gravity of xylene ensured that palladium ions would not enter the inner tube of CNTs. The test results and the characterization of the materials showed that Fe@CNTs@Pd is a promising material for highly efficient TCE remediation. It is acknowledged that the application of this material is still limited by its high cost due to the price of Pd. In future work, we will look into alternative low-cost catalysts to replace Pd for new synthesized composites.

## Supplementary Material

XPS spectrum of scan survey of Fe@CNTs@Pd (a), C1s high-resolution spectrum of Fe@CNTs@Pd (b).

## Supplementary Material

N2 adsorption and desorption isotherms and size distribution (inset) of unmodified CNTs (a), blank CNTs (b), Fe/Pd@CNTs (c), Fe@CNTs@Pd (d)

## Data accessibility

The datasets supporting this article have been uploaded as part of the electronic supplementary material.
