# Supplementary material for: Preparation of palladized carbon nanotubes encapsulated iron composites: highly efficient dechlorination for trichloroethylene and low corrosion of nanoiron
Source: R Soc Open Sci. 2018 Jun 27;5(6):172242. doi: 10.1098/rsos.172242 (PMC6030302; doi:10.1098/rsos.172242)
Supplement: N2 adsorption and desorption isotherms and size distribution (inset) of unmodified CNTs (a), blank CNTs (b), Fe/Pd@CNTs (c), Fe@CNTs@Pd (d) [file rsos172242supp2.docx]

**Preparation of Palladized Carbon Nanotubes Encapsulated Iron** **Composites: Highly Efficient Dechlorination for TCE and Low Corrosion of Nanoiron**

**Xinyu Wang^1^, Wei Wang^1^, Greg Lowry^2^,Xiaoyan Li^2^,** **Yajie Guo^2^, Tielong Li^1,2^^[[1]](#footnote-1)^***

*1. College of Environmental Science and Engineering/Tianjin Key Laboratory of Environmental Remediation and Pollution Control/Ministry of Education Key Laboratory of Pollution Processes and Environmental Criteria, Nankai University,Wei Jin Road 94, Tianjin 300071, China*

*2. Department of Civil & Environmental Engineering, Carnegie Mellon University, Pittsburgh, Pennsylvania 15213, United States*


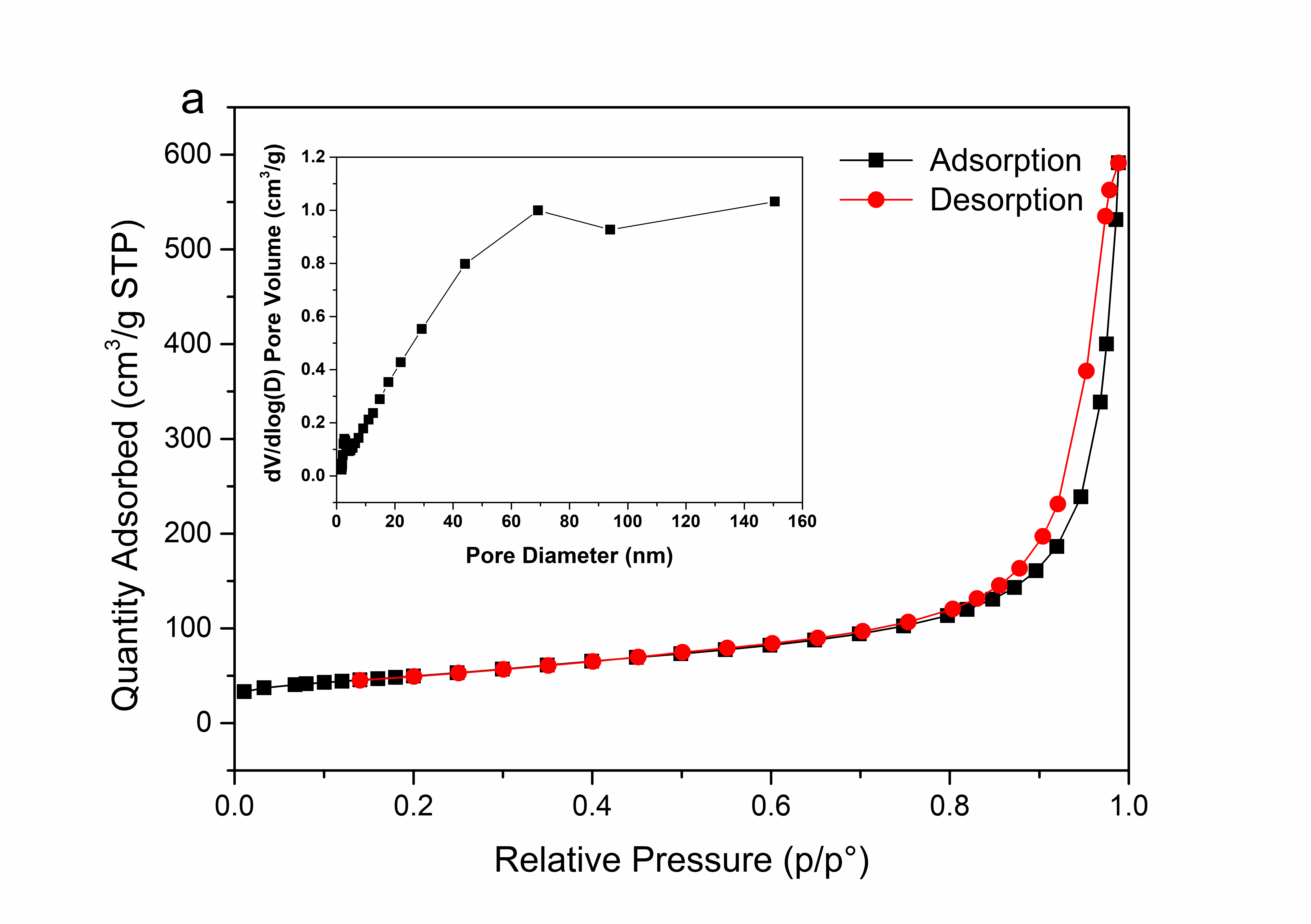


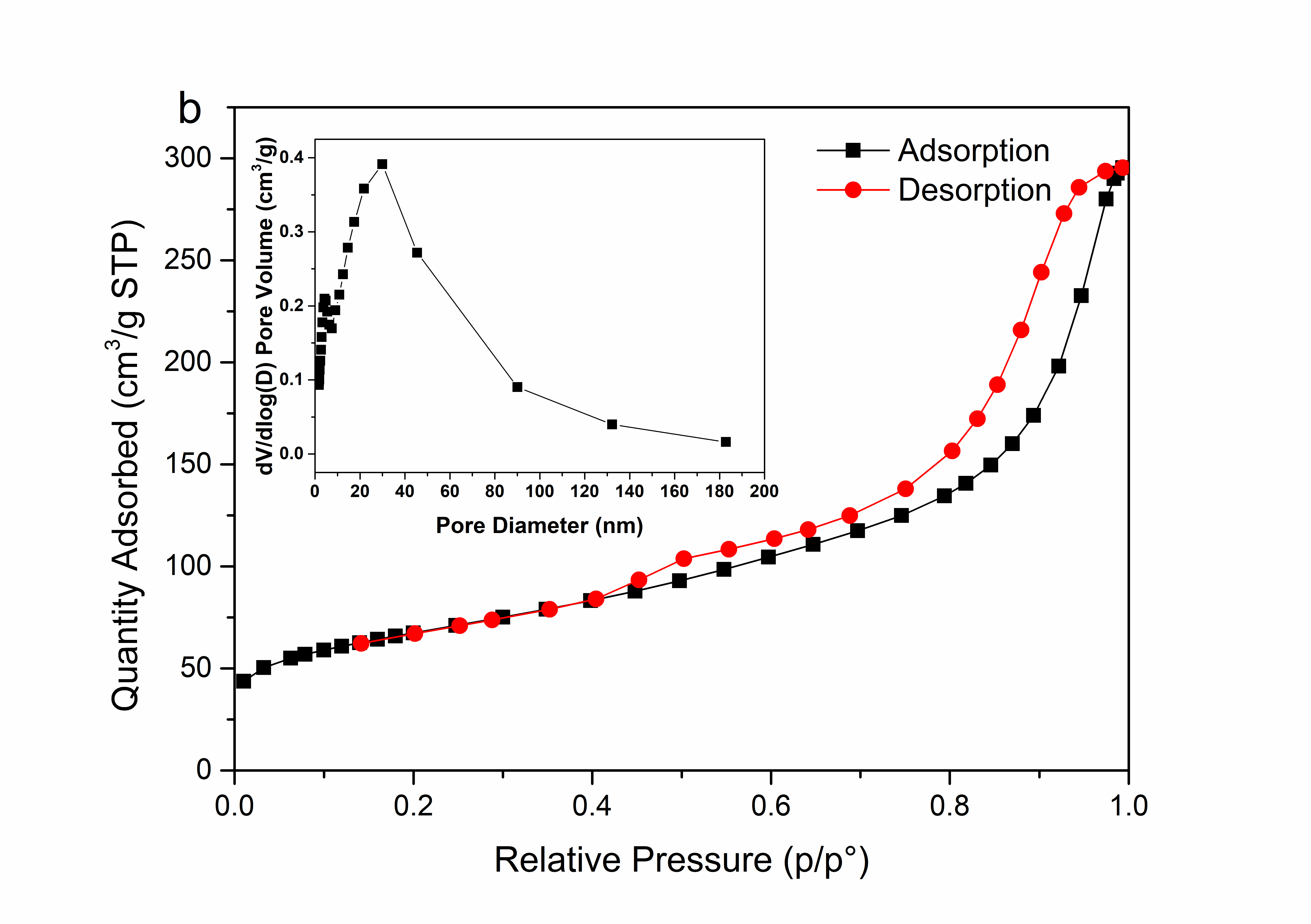


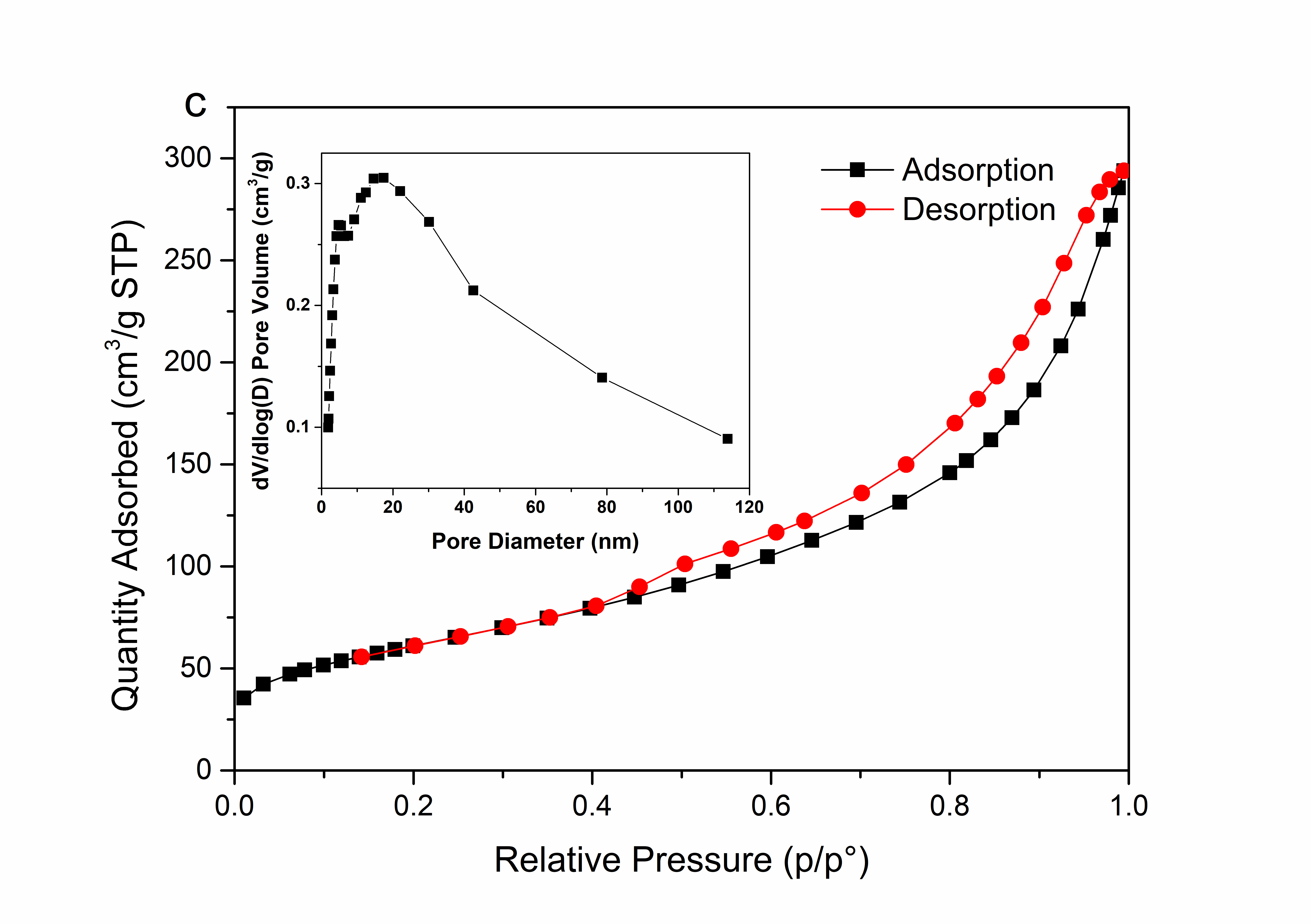


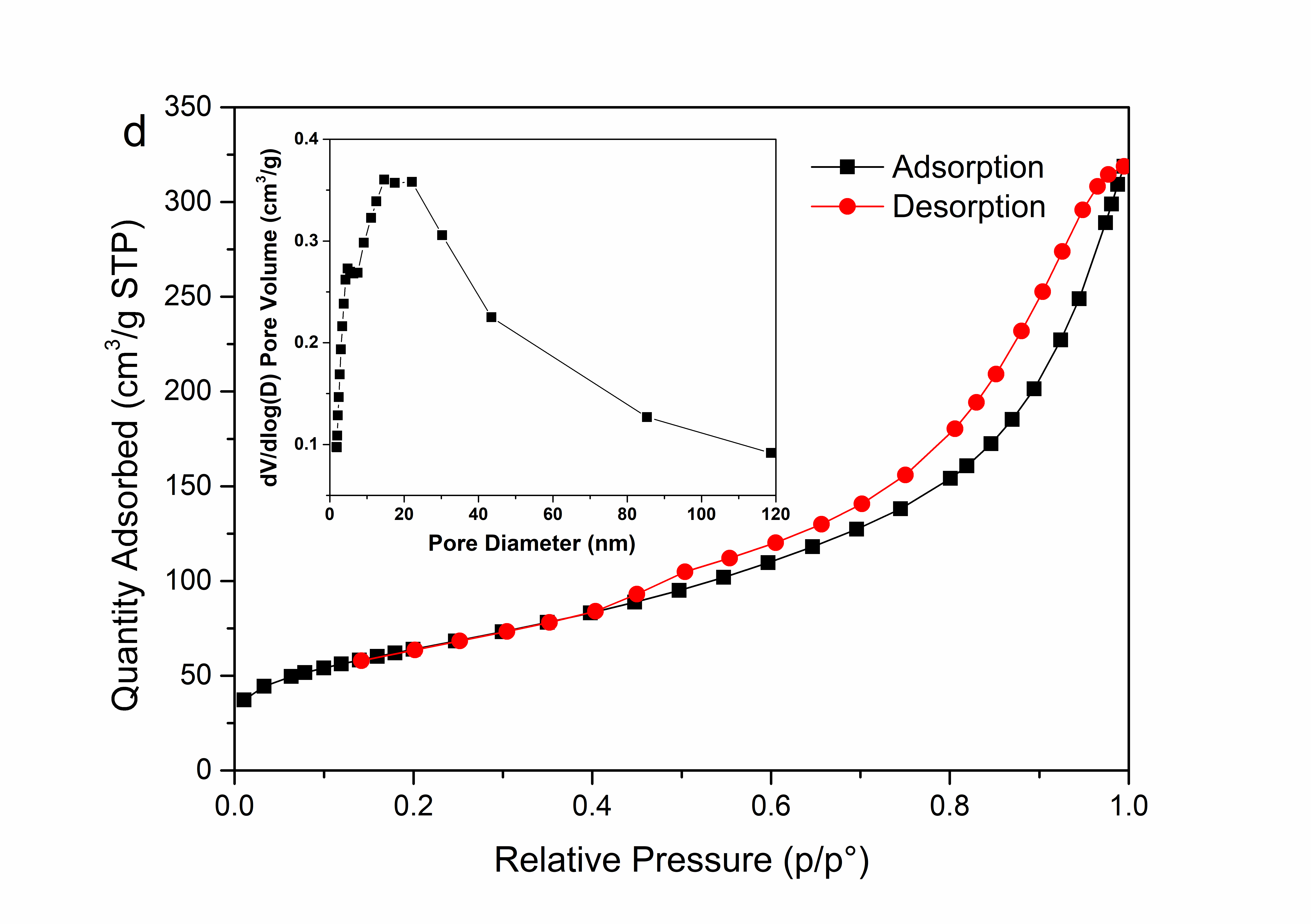


**Figure. S2 N2 adsorption and desorption isotherms and size distribution (inset) of un****modified CNTs (a),** **blank CNTs (b), Fe/Pd@CNTs (c), Fe@CNTs@Pd (d)**

1. * Corresponding author e-mail: [litielong@nankai.edu.cn](mailto:litielong@nankai.edu.cn).

   Address: College of Environmental Science and Engineering, Nankai University, 94 Weijin Street, Tianjin, 300071, China. [↑](#footnote-ref-1)
